# Health behaviors and social determinants of health in children from under-resourced communities: does weight status play a role?

**DOI:** 10.3389/fspor.2025.1695539

**Published:** 2026-01-05

**Authors:** Paul Son, Yuxin Nie, Qiaoyin Tan, Amanda E. Staiano, Fahui Wang, Gang Hu, Stewart Gordon, Peyton Murray, Cehong Luo, Yutian Zeng, Renee A. Underwood, Senlin Chen

**Affiliations:** 1 School of Kinesiology, Louisiana State University, Baton Rouge, LA, United States; 2 Pediatric Obesity and Health Behavior Laboratory, Pennington Biomedical Research Center, Baton Rouge, LA, United States; 3 Department of Geography & Anthropology, Louisiana State University, Baton Rouge, LA, United States; 4 Chronic Disease Epidemiology Lab, Pennington Biomedical Research Center, Baton Rouge, LA, United States; 5 Louisiana Healthcare Connections, Baton Rouge, LA, United States; 6 Department of Kinesiology and Health Studies, Southeastern Louisiana University, Hammond, LA, United States

**Keywords:** childhood obesity, health behavior, health disparities, social determinants of health, weight status

## Abstract

**Background/objectives:**

Social determinants of health (SDOHs) may affect children's health and health behaviors. This study aimed to understand the relationship between health behaviors and SDOHs in a child population from under-resourced communities [i.e., Medicaid eligible or enrolled, overweight or with obesity (OWOB), predominantly Black or African Americans].

**Methods:**

Following a stratified sampling strategy, parent proxies (*N* = 311) completed an online survey to measure participants (5–12 years old) health behaviors and SDOHs including socioeconomic status [SES; Area Deprivation Index (ADI), household income], living conditions, and food insecurity.

**Results:**

Participants with OWOB showed greater screen time than normal weight (NW) children. Health behaviors (i.e., physical activity, screen time, sleep, dietary behavior) generally favored the higher SES group (considering household income and ADI). SDOHs (as a variant) correlated with health behaviors (the other variant; Canonical *r* = 0.27, *p* < 0.05). Of the SDOHs, household chaos negatively correlated with regular bedtime routines in both weight status groups (NW: *r* = −0.19, *p* < 0.05; OWOB: *r* = −0.24, *p* < 0.01). Adverse living conditions and greater food insecurity were associated with more screen time and, unexpectedly associated with more physical activity (*r* ranged from 0.19 to 0.22, *p* < 0.05) in NW participants.

**Conclusions:**

The findings unraveled differences in health behaviors by weight status and SDOHs. SDOHs showed significant correlations with health behaviors, and these correlations were slightly greater in NW children. Weight status plays an important role in the relationship between health behaviors and SDOHs among children from under-resourced communities.

## 1 Introduction

Social determinants of health (SDOHs) may affect children's health behaviors (1). SDOH refers to environmental, non-medical factors that influence health, including economic stability, healthcare access, education, neighborhood and built environment, and social community context (2). SDOHs are important factors to consider when addressing health disparities across communities and populations. Underprivileged populations from lower socioeconomic status (SES) households and impoverished communities face many life challenges such as poverty, health illiteracy, inadequate health insurance coverage, and low healthcare accessibility, making them more vulnerable to chronic diseases and poor health conditions (3). Because of these challenges, children from under-resourced communities are at elevated risk for obesity, cardiovascular disease, type 2 diabetes, or other health problems (4, 5). To address health disparities and promote health equity, it is important to understand the relationship between SDOHs, and health behaviors, and consequently health outcomes in underprivileged children (6–9).

Children from under-resourced households have limited opportunities to adopt and sustain health-enhancing behaviors, which may affect their overall health. To achieve optimal health, children are recommended to be physically active (≥ 60 min of moderate-to-vigorous physical activity per day), reduce exposure to electronic screens (≤ 2 h per day; especially those in sedentary posture), have balanced meals in moderation (e.g., 5 or more servings of fruits and vegetables per day), and maintain healthy sleep hygiene (9–11 h uninterrupted sleep per day with bedtime routines) (10, 11). Compared to children from affluent families, children from lower SES households (e.g., lower income, greater household chaos) are less likely to meet these behavioral guidelines (12–14). Limited resources and additional home factors such as living conditions (e.g., housing condition and quality, utilities, access to childcare and education) and food insecurity may directly strain children's healthy development and health-enhancing behaviors. Children in food-insecure households tend to experience poor physical and mental health, higher hospitalization rates, and a higher possibility of developmental and educational delays compared to their peers (15). While food insecurity may not directly correlate with an increased risk of obesity during childhood, deprivation in childhood may be linked to adulthood obesity (16). Beyond the household, adverse neighborhood and community characteristics (e.g., high crime rate, no access to green space, non-walkable or -bikeable streets) may further impact children's health behaviors and health status (17, 18). Area Deprivation Index (ADI), a multidimensional measure of neighborhood socioeconomic disadvantages considering income, education, employment, and housing quality, is a widely recognized measure affecting individual and community health outcomes (19). Although prior research has examined the relationship between factors related to ADI and some health behaviors (20), no prior studies have empirically tested how ADI as a multidimensional construct correlated with multiple health behaviors (physical activity, screen time, diet, and sleep) among children from under-resourced communities. Since individual-level SES measures cannot fully capture environmental barriers or facilitators, incorporating ADI allows researchers to assess how broader structural and neighborhood conditions constrain or enable healthy behaviors.

Over a third of children are overweight or with obesity (OWOB) in the United States, and the obesity prevalence is higher in under-resourced communities, especially in deep south states (e.g., Louisiana), demanding disparity-focused obesity interventions. Such interventions may become more effective when influential personal and environmental factors are considered for the priority population. Prior research indicates OWOB children often display different behavioral habit patterns than normal weight (NW) children, and children's weight status is found to be associated with physical inactivity and sedentary habits (21, 22). Compared to the NW children, OWOB children are usually less physically active (23–25). They also have less healthy dietary habits, such as greater intake of ultra-processed foods, lower consumption of natural juices, and difficulty maintaining a balanced diet (21, 26, 27). Additionally, obesity significantly increases the risk of having a sleep disorder such as obstructed sleep apnea (28, 29). As a result, OWOB children are less likely to have restful and healthy sleep than NW children (30). Furthermore, OWOB children aged 4–11 were found to be more likely to exceed screen time recommendations compared to NW children (31).

Of the four health behaviors focused on this study (i.e., physical activity, screen time, sleep, dietary behavior), screen time has recently received significant research attention, yet accurately measuring screen time continues to be a challenge. Screen time refers to time spent using a device such as a computer, television, or games console (32). As a health risk factor, screen time is often discussed in a similar context as sedentary behavior [i.e., any waking behavior characterized by an energy expenditure ≤ 1.5METs, while in a sitting or reclining posture (33)]. However, screen time is not unidimensional or homogenous, as it may include behaviors in passive or interactive modes and/or in sedentary or standing posture (34). Trost et al. (35) developed the Movement Behaviors Questionnaire (MBQ), which included a set of questions to measure passive and interactive screen time in sedentary and standing postures, separately, in babies (MBQ-B) and children (MBQ-C) populations (35). Limited prior research has measured children's screen time through this new approach, nor explored its relationship with weight status and SDOHs.

To fill the research gaps, this study aims to explore the relationship between health behaviors and SDOHs based on weight status in a largely underprivileged child population. Specifically, the study has three research purposes: 1) to determine if health behaviors differ by weight status and SES (household income and community-level socioeconomic disadvantage), 2) to examine the relationship between health behaviors and SDOHs, and 3) to explore if these relationships differ by weight status. Findings from this study have the potential to guide the design of tailored childhood obesity intervention strategies to mitigate health disparities in children from under-resourced communities.

## 2 Method

### 2.1 Research design, setting and participants

This study conducted cross-sectional comparisons of health behaviors and examined the relationship between health behaviors and SDOHs by weight status. A stratified sampling strategy was utilized to recruit a representative sample of elementary schools in the Baton Rouge Metropolitan Statistical Area (BRMSA), Louisiana. Schools were stratified into community types considering urbanicity and ADI score ranges. The BRMSA includes ten diverse parishes (counties) with many health disparities. As shown in Table 1, we categorized the sampling region into nine types of communities based on ADI and urbanicity. ADI ranges from 1 to 10, with higher scores representing a greater disadvantage. We only targeted areas with medium to high socioeconomic disadvantage, excluding areas with ADI values lower than 5. ADI values from 5 to 10 were categorized into three groups (5–6, 7–8, 9–10). In the analytical process, ADI was recoded into two groups (lower ADI: 1–6; higher ADI: 6.1–10) since this approach provides clearer and more interpretable contrasts between levels of community disadvantage. According to the U.S. Census, urbanicity has three categories of locales including urban, high-density (suburban), and rural (36). We used local schools to recruit parents of students attending the schools. Given the varying number of schools across community types (e.g., more urban schools with high ADI scores), we randomly selected 1–3 schools per community type to recruit 18 schools in total (i.e., 40 children per school stratified by gender and grade). Back-up schools with similar ADI score and urbanicity classification were included in the event of decline or withdrawal from participation (Table 1). The study was approved by the institutional review boards (IRBs) of Louisiana State University and Pennington Biomedical Research Center. Parent participants completed consent electronically, before completing the content survey.

**Table 1:** Distribution of public elementary schools across nine types of communities.

		Urbanicity categories		
		Urban area	Low-density area	Rural area
ADI Score Range	9–10	11 (2)^a^	1 (1)^a^	3 (2)^a^
	7–8	15 (3)^a^	3 (2)^a^	7 (2)^a^
	5–6	9 (2)^a^	5 (2)^a^	8 (2)^a^

a The first number represents the number of eligible schools, and the number inside the parentheses represents the schools recruited.

Invitations of participation in the survey were sent to 26 elementary schools within the 10-parish BRMSA area. After multiple attempts, 13 schools agreed to participate. Each parent participant was offered a $20 gift card as an incentive to take the survey. Of the 543 parents who began the survey, 331 completed the survey. After the removal of duplicates and unusable surveys, the final valid sample size was 311. Because recruitment was based on the availability of schools in the state that represent community types by ADI and urbanicity, no formal power calculation was conducted. However, the final analytic sample (*n* = 311) provides sufficient variability across weight status and SES categories for the planned cross-sectional comparisons.

### 2.2 Variables and measures

#### 2.2.1 Health behaviors

Four health behaviors including physical activity, sedentary behavior, sleep, and dietary behavior were measured through a survey. For physical activity, parent proxies were asked to report the number of days their child was physically active for at least 60 min per day during the past 7 days. This question was adopted from the National Health and Nutrition Examination Survey (NHANES) (37). Additionally, the Godin-Shepard exercise questionnaire was administered to measure physical activity including strenuous, moderate, and mild-intensity exercises. Parents were asked, “During the past week, how often did your child engage in any activity long enough to work up a sweat during their leisure time?” The Godin-Shepard LSI index was calculated after considering intensity and time (number of 15-minute segments): 9×Strenuous+5×Moderate+3×Light (38). The questionnaire has been widely used to measure physical activity in various populations for its acceptable validity and reliability (39, 40).

To measure screen time, parent proxies reported their child's electronic screen device use in minutes and hours over an average 24-hour period to questions, adopted from the Movement Behaviors Questionnaires – Children (MBQ-C) survey. Questions related to screen time (1. passive screen time: viewing TV, videos/internet clips, or movies; 2. interactive screen time: playing games, looking at photos, or video chatting [e.g., FaceTime, Zoom, Skype] on a screen-based device such as a computer or laptop, video game console, iPad, tablet, or smartphone) were repeatedly asked for weekdays vs. weekend days, in sedentary vs. standing postures, and interactive vs. passive screen time. Scoring instructions of the MBQ-C were closely followed to obtain weighted average scores for screen time (sedentary vs. standing, interactive vs. passive, total) for subsequent data analysis. The MBQ-C was recently validated using parents and young children's dads (35).

To assess children's sleep, parent proxies completed the modified short-form version of the Children's Sleep Habits Questionnaire (41). Questions included regularity of sleep [e.g., whether their child went to bed at around the same time (42)] and duration of sleep based on the usual amount of overnight and daytime (naps) sleep with options in 1-hour increments during the weekdays and weekend days. The weighted average daily sleep time was calculated by adding 5 times the average sleep time during weekdays and 2 times the average sleep time during weekends, then dividing the total by 7. Lastly, parents were asked about their child's consumption of fruits and vegetables per day. They reported the number of servings of fruits and vegetables their child ate per day over the past week.

#### 2.2.2 Social determinants of health

We measured the participants' SES (including household income and Area Deprivation Index [ADI]), household chaos, living conditions, and food insecurity. Parents reported the combined annual household income, where they selected one of the choices such as “less than $10,000” or “$10,000 to $29,000”. They also reported their home address zip code, which was used to look up the ADI value. ADI value is categorized based on zip codes of housing units from the 2020 Census data. It is weighted by the size of each block group within the corresponding zip code. ADI categories were coded as 1 for low deprivation (ADI = 1–6) and 2 for high deprivation (ADI = 6.1 through 10).

To assess living conditions, parent proxies were asked to respond to questions regarding utility (e.g., “In the past 12 months has the electric, gas, oil, or water company threatened to shut off services in your home?”), childcare accessibility (e.g., “Do problems getting childcare make it difficult for you to work or study?”), and housing condition and quality (e.g., “In the last month, have you had concerns about the conditions and quality of your housing?”). Parent proxies also responded to a few questions related to food insecurity (e.g., “In the last 12 months, did you ever eat less than you felt you should because there wasn't enough money for food?”) (43). Additional questions addressed access to free food, such as free groceries from a food pantry, food bank, or church, and free meals from a church, shelter, or home-delivered meal service. Each question was coded as 1 (yes) or 0 (no), and the responses were summed to calculate the level of food insecurity.

To assess household chaos, the Confusion, Hubbub, and Order Scale (CHAOS) was adopted and administered in the survey (1). This scale reflected elements of household chaos and disruption, excessive noise, mess, lack of organization, and hectic or frantic activities. The scale included 14 true and false questions. An example of an item was: “It's so noisy, you can't hear yourself think in our home.” A sum score was computed to indicate the level of household chaos, with higher scores indicating a more chaotic home environment.

### 2.3 Data collection

Data collection began with recruitment of parents of children (October 2023 – July 2024) attending the target schools within our sampling region and community categories. Recruitment emails were sent to administrators in school districts and schools (superintendents and principals), followed by weekly reminder emails and then phone calls. The research team visited the schools and met with administrators. Each participating school was offered a $500 gift card to coordinate the survey distribution to parents. Each school appointed a contact person to handle the communication between the research team and parents until the sampling goal and response count were attained. A survey including the consent form, sociodemographic questions, and questions for health behaviors and SDOH was created and administered through the Research Electronic Data Capture (REDCap) platform for data collection (44, 45). The survey link was distributed to the contact persons at the target schools who subsequently invited parents to take the survey.

### 2.4 Data analysis

Data were downloaded from the REDCap onto an Excel file for cleaning and processing. We removed extreme outliers from the dataset based on visual inspection and normality check: total screen time≥720 min/day, fruit and vegetable servings ≥17/day, Godin-Shepard LSI score ≥ 433, and Sleep (average) > 852 min/day. We also removed BMIz scores that were ≥4 or ≤ −4, when conducting cross-sectional comparison based on weight status. In addition, to compare NW and OWOB groups, we did not include BMI percentile scores <5% (i.e., underweight) in the analysis. The processed data were subsequently imported into the SPSS for analysis.

Descriptive (frequencies, central tendency, and variability) and inferential statistical analyses were conducted to address the research questions. Analyses of variance (ANOVAs) were implemented to determine the differences in health behaviors by weight status and SES. Then, canonical correlation and Pearson correlational analyses were completed to examine the relationship between health behaviors and SDOHs. We next separated the correlational analysis by weight status, to explore if this relationship between health behaviors and SDOHs would show different patterns between participants with NW vs. OWOB. Partial eta squares were reported as effect sizes for ANOVAs with a significance level set at 0.05. Significant cross-sectional differences were visualized using bar charts. To ensure the validity of the analyses, we examined the statistical assumptions of ANOVA, Pearson correlation, and canonical correlation. Normality was assessed using histograms, skewness and kurtosis values, and visual inspection of Q-Q plots. Homogeneity of variance for ANOVA models was evaluated using the Levene's test. Multicollinearity were inspected through scatterplot matrices and correlation matrices before performing Pearson and canonical correlation analyses. Bonferroni-adjusted *post hoc* tests were performed following the one-way ANOVAs, to identify specific group differences while controlling Type I error. These diagnostics indicated that the assumptions were adequately met for our analyses.

## 3 Results

Table 2 presents the sociodemographic characteristics of study participants. Of all respondents, the vast majority were women (most likely mothers) providing data for their children. If more than one child was attending an elementary school in the same household, the parent was asked to complete the survey on behalf of their oldest child. The children had a roughly even gender split, most of whom were Black (71.38%) or non-Hispanics (94.86%). The sample consisted of children attending kindergarten through sixth grade. Most of the participants (or their families) were eligible for Medicaid (82.64%) and enrolled in Medicaid (83.60%); while more than half were OWOB (50.32%). A large percentage of the participants came from low-income households, with 42.44% reporting less than $30,000 annual household income. The average ADI value across the communities was 5.83 (±2.00). We split the sample into lower ADI (48.2%) and higher ADI (51.8%) groups for subsequent analysis.

**Table 2:** Sociodemographic characteristics of the sample.

Sociodemographic variables	Categories	*N*	%
Sex (Parent)	Men	32	10.32
	Women	278	89.68
Sex (Child)	Boys	160	51.45
	Girls	151	48.55
Race	White	64	20.58
	Black	222	71.38
	American Indian or Alaska Native	1	0.32
	Asian	7	2.25
	Native Hawaiian	0	0.00
	Pacific Islander	3	0.96
	Multi-race	14	4.50
	Other	0	0.00
Ethnicity	Non-Hispanic	295	94.86
	Hispanics	16	5.14
Grade	K	50	16.08
	1st	48	15.43
	2nd	42	13.50
	3rd	63	20.26
	4th	50	16.08
	5th	43	13.83
	6th	15	4.82
Weight status	Underweight (<5%)	35	11.29
	Normal weight	119	38.39
	Overweight or obese	156	50.32
Medicaid eligible	Yes	257	82.64
	No	36	11.58
	Not Sure	18	5.79
Medicaid enrolled	Yes	260	83.60
	No	44	14.15
	Not Sure	7	2.25
Household income	Less than $10,000	55	17.68
	$10,000–$29,999	77	24.76
	$30,000–$49,999	71	22.83
	$50,000–$69,999	41	13.18
	$70,000–$89,999	16	5.14
	$90,000–$109,000	7	2.25
	$110,000–$139,000	6	1.93
	$140,000 and above	5	1.61
	Prefer not to answer	33	10.61
Area deprivation index	Lower ADI (1–6)	148	48.2
	Higher ADI (6.1–10)	159	51.8

Table 3 shows the descriptive results of the health behaviors for the sample, including physical activity, screen time, sleep, and diet. Parents reported that their children, on average, met the recommended physical activity time on more than four days per week (46), with the LSI index being 71.46. In contrast, parents reported that children accrued nearly six hours of screen time per day, mostly in a sedentary posture. Daily average passive screen time exceeded interactive screen time. For sleep, parents reported that their child had regular bed routines on 3.21 days per week, although they averaged 9.81 h of sleep per night with 75.2% meeting the sleep guideline (i.e., 9–11 h per day). They slept 40 min/day more on weekend days than weekdays. In addition, their daily average intake of fruits and vegetables was 4.44 servings, with 38.5% meeting the guideline (i.e., 5 or more servings per day).

**Table 3:** Parent-reported child health behaviors.

Health behaviors	N	Min	Max	M	SD
Physical activity					
Godin-Shepard Leisure Score Index (LSI)	310	0.00	238.00	71.46	46.89
# of active days in a week (day)	302	0.00	7.00	4.12	2.50
Screen time (daily)					
Passive screen time (min)	253	0.00	651.43	219.29	113.66
Sedentary (min)	253	0.00	570.00	144.09	98.90
Standing (min)	253	0.00	339.86	75.20	72.85
Interactive screen time (min)	253	0.00	596.00	120.74	103.65
Sedentary (min)	253	0.00	480.00	73.88	79.55
Standing (min)	253	0.00	596.00	46.86	63.99
Total screen time (min)	253	10.00	713.57	340.03	170.90
Sedentary (min)	253	0.00	672.86	217.97	141.09
Standing (min)	253	0.00	692.00	122.06	112.89
Sleep					
Regular bed routine in a week (day)	309	0.00	4.00	3.21	1.04
Sleep duration – daily average (min)	303	336.43	847.14	588.66	55.28
Sleep duration on weekday (min)	304	315.00	1,350.00	579.05	73.55
Sleep duration on weekend day (min)	305	60.00	1,320.00	620.89	116.54
Diet					
Fruits and vegetables intake (serving)	304	0.00	14.00	4.44	2.59
Daily fruits intake (serving)	304	0.00	11.00	2.53	1.59
Daily vegetables intake (serving)	304	0.00	12.00	1.91	1.49

**Figure 1:**
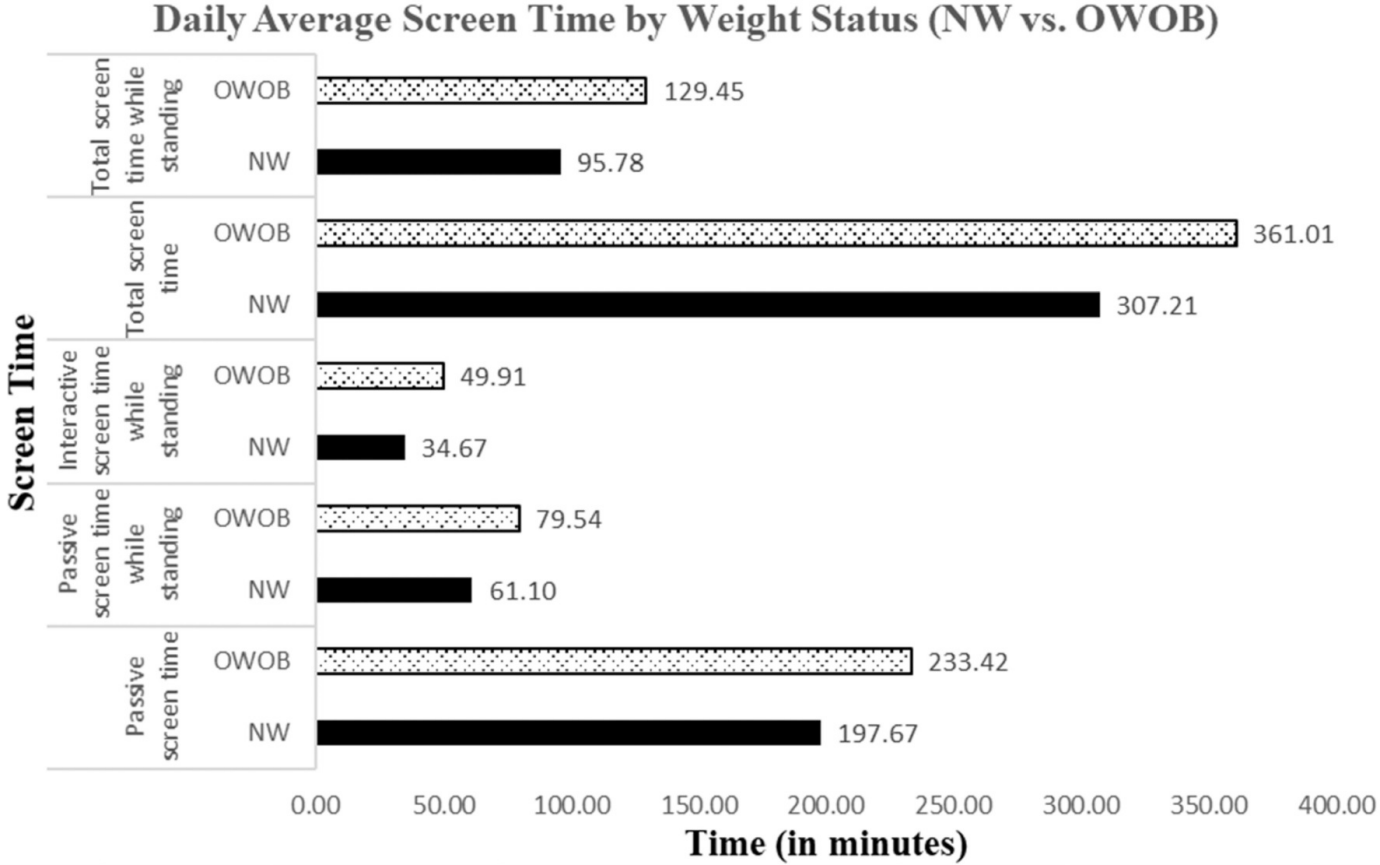
Daily screen time by weight status.

**Figure 2:**
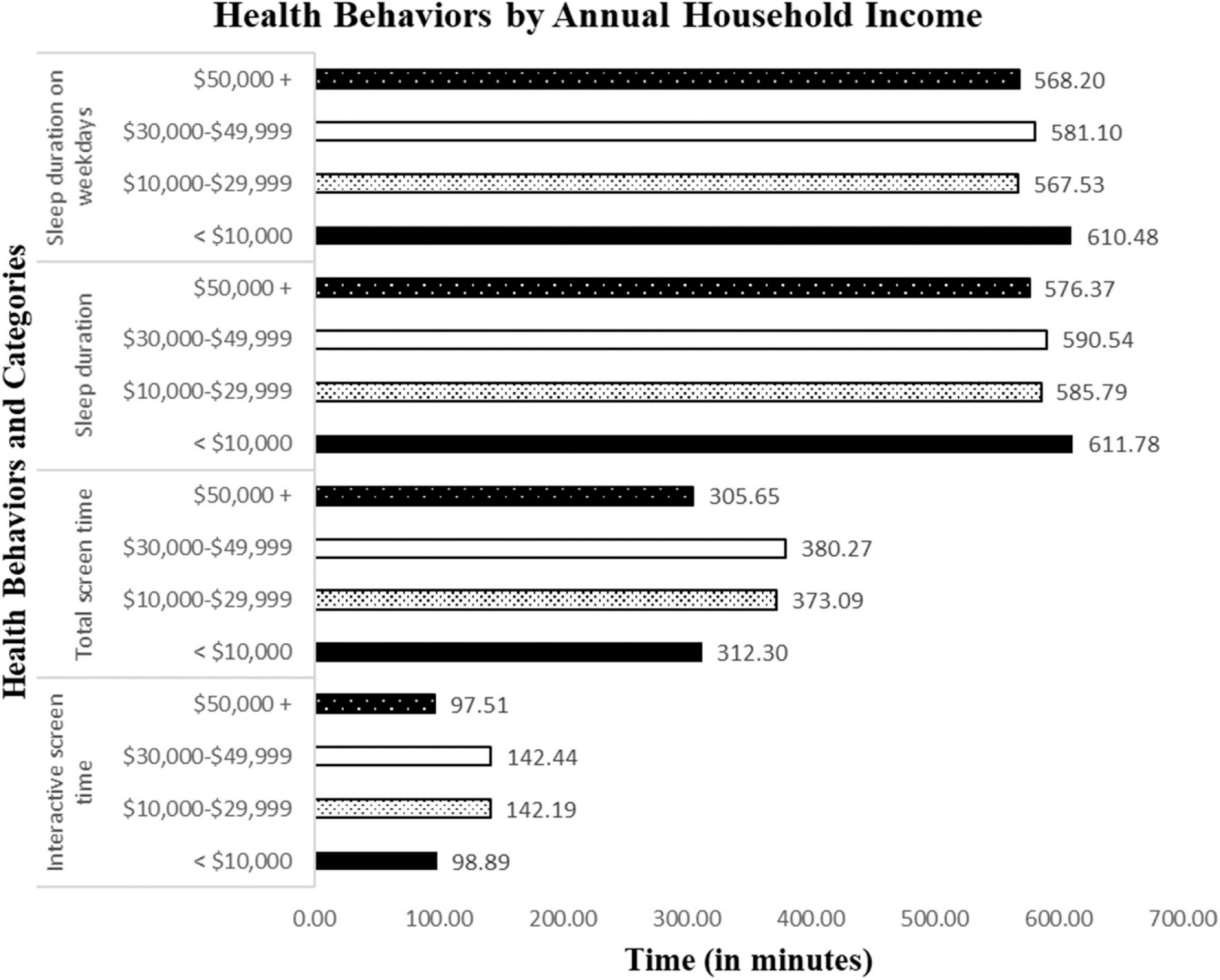
Sleep and screen time by annual household income.

Of the four health behaviors, screen time showed significant group differences by weight status. Specifically, compared to those with NW, parents of OWOB children reported that their children had significantly more passive screen time (*F*_1,214_ = 5.70, *p* < .05, $\eta_{p}^{2}=0.03$), passive screen time while standing (*F*_1,214_ = 4.06, *p* < .05, $\eta_{p}^{2}=0.02$), interactive screen time while standing (*F*_1,214_ = 4.53, *p* < .05, $\eta_{p}^{2}=0.02$), total screen time (*F*_1,214_ = 5.57, *p* < .05, $\eta_{p}^{2}=0.03$), and total screen time while standing (*F*_1,214_ = 5.92, *p* < .05, $\eta_{p}^{2}=0.03$). Figure 1 illustrates the group differences in these screen time variables. In addition, screen time and sleep differed by annual household income: interactive screen time (*F*_3,222_ = 3.53, *p* < .05, $\eta_{p}^{2}=0.05$), total screen time (*F*_3,222_ = 3.10, *p* < .05, $\eta_{p}^{2}=0.04$), sleep duration (*F*_3,267_ = 4.33, *p* < .01, $\eta_{p}^{2}=0.05$), and sleep duration on weekdays (*F*_3,268_ = 4.42, *p* < .05, $\eta_{p}^{2}=0.05$). Figure 2 illustrates the mean estimates of these behaviors by annual household income. The highest and lowest income groups showed less screen time exposure than the two middle-income groups. As for sleep, all income groups showed adequate daily average sleep durations (>9 h daily average), although the lowest income group showed the longest sleep daily average (overall and on weekdays).

**Figure 3:**
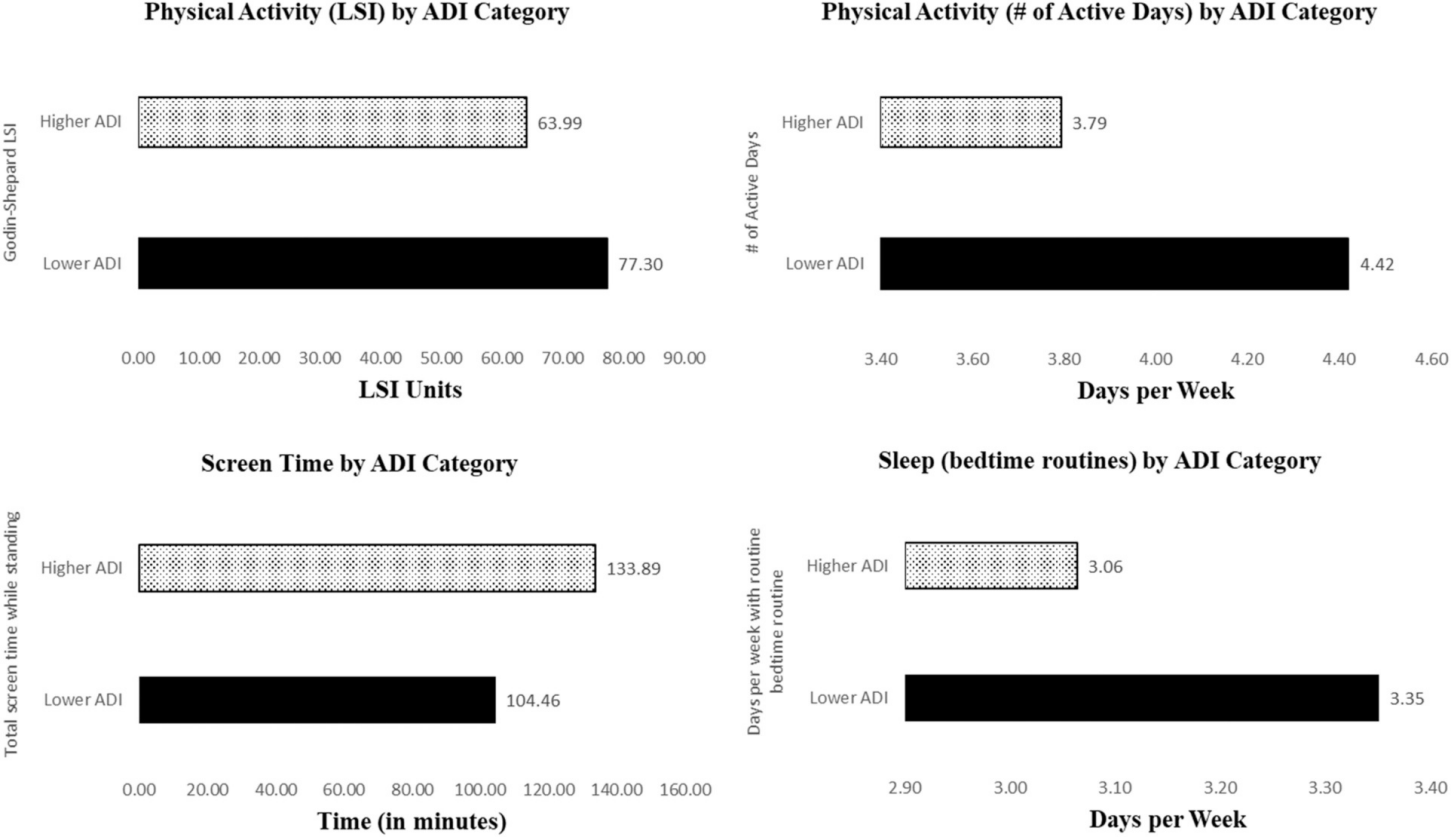
Difference in physical activity (LSI^1^ and number of active days), screen time, and sleep between lower-ADI^2^ and higher-ADI group. Higher ADI indicates more socioeconomic disadvantage, and lower ADI shows less socioeconomic disadvantage.

Furthermore, we further examined whether health behaviors differed between socioeconomically less disadvantaged (lower ADI category) and more disadvantaged communities (higher ADI category). Physical activity, screen time, and sleep favored participants residing in the less socioeconomically disadvantaged communities. Specifically, Godin-Shepard LSI (*F*_1,304_ = 6.55, *p* < .05, $\eta_{p}^{2}=0.02$), the number of active days per week (*F*_1,296_ = 4.70, *p* < .05, $\eta_{p}^{2}=0.02$), total screen time while standing (*F*_1,247_ = 4.44, *p* < .05, $\eta_{p}^{2}=0.02)$, and days per week with regular bedtime routines (*F*_1,303_ = 5.91, *p* < .05, $\eta_{p}^{2}=0.02$). Figure 3 illustrates these mean differences.

Lastly, our canonical correlation analysis revealed that environment including living conditions, food insecurity, and household chaos showed a significant correlation with health behaviors (canonical *r* = 0.27, *p* < 0.05). Pearson's correlation analysis was conducted to further identify the specific relationship between health behaviors and environment, we performed Pearson's correlation analysis, separately for NW and OWOB groups.

The correlation matrix included in the Supplementary Table A1 summarizes the correlation coefficients among the variables. Regarding the correlation coefficients, household chaos negatively correlated with the number of days per week with regular bedtime routines for both groups (NW: *r* = −0.19, *p* < 0.05; OWOB: *r* = −0.24, *p* < 0.01). For participants with NW, living conditions positively correlated with total screen time (*r* = 0.22, *p* < 0.05), interactive screen time (*r* = 0.22, *p* < 0.05), and interactive screen time while standing (*r* = 0.21, *p* < 0.05). Food insecurity showed an unexpected positive correlation with physical activity (i.e., Godin-Shepard LSI index; *r* = 0.19, *p* < 0.05). While these bivariate correlations had values around 0.2 or −0.2, they were statistically significant.

## 4 Discussion

The purpose of this study was 1) to determine if children's health behaviors differ by weight status and SES (both household income and community-level socioeconomic disadvantage), 2) to examine the relationship between health behaviors and SDOHs, and 3) to explore if these relationships differ by weight status. Our analyses revealed interesting findings discussed below.

Health behaviors presented differential patterns by weight status, income level, and ADI. Compared to NW children, OWOB children engaged in significantly higher amounts of passive and interactive screen time while standing, as well as more total screen time and total passive screen time. These findings are consistent with Anderson et al.'s observation that OWOB children aged four to eleven were more likely to have over two hours of screen time per day than NW children (31). The literature suggests that excessive screen time is detrimental to health and adds the risk of obesity (47). The child sample in the present study showed over five hours of screen time on average per day, which is very concerning. The higher amount of screen time observed in OWOB children suggests that future childhood obesity prevention interventions should target modifiable factors to reduce children's screen time (passive and interactive screen time while standing, in particular). Prior research has shown that screen time exposure while eating is associated with increased calorie intake and sleep disruption, which, along with screen time *per se*, may contribute to weight gain (48, 49).

Screen time (interactive screen time and total screen time) and sleep (daily average duration and average duration on weekdays) showed some group differences by household income. The highest ($50,000 or higher annually) and lowest income (<$10,000 annually) groups showed less screen time exposure than the two middle-income groups. Previous research found that household income was a predictor of digital technology use among school-aged children (50). While the root cause of this difference in screen time by household income is unknown, we speculate that the lower screen time reported in the lowest income group could be related to the limited access to portable electronic devices such as tablets and smartphones; whereas, the lower screen time reported in the higher income group may be due to higher parental control of screen use and improved access to other extracurricular activities. As for sleep behavior, all income groups showed adequate daily average sleep durations (>9 h daily average), although the lowest income group (< $10,000 annually) showed the longest sleep daily average (overall and on weekdays). This finding is inconsistent with Hawkins et al.'s observation, as living in a low-income household does not necessarily directly disrupt children's sleep (14). However, Kim et al. (2025) suggested that financial difficulties could affect sleep either positively or negatively, as the relationship between household income and sleep is not simply linear (51).

To further explore the relationship between health behaviors and SES, we examined the role of community deprivation. Our study found that children living in higher ADI communities (more disadvantaged) showed lower physical activity (lower LSI, fewer active days per week), more screen time, and less regular bedtime routines than children living in the lower ADI neighborhoods (less disadvantaged). Such results are consistent with previous research findings which indicate socioeconomically disadvantaged children face greater barriers to engaging in health-promoting behaviors, due to factors such as limited access to safe recreational space (52, 53). This set of findings suggest that ADI is a crucial community-level SES factor to consider when addressing health disparities.

To address our second and third research purposes, we further investigated the relationship between health behaviors and SDOHs (household chaos, living conditions, food insecurity). Statistically significant correlations were observed in our canonical correlation (7.1% of the variances accounted for by SDOHs) and Pearson correlational analyses. We also found the correlational relationship between health behaviors and SDOHs to be slightly stronger in NW children than in OWOB children. Household chaos, living conditions, and food insecurity are known SDOHs that affect our daily living and health status. However, few prior studies have examined the relationship between SDOHs and health behaviors. A rare exception was Kracht et al. that reported that young children living in homes with high levels of household chaos were less likely to meet movement behavior guidelines compared to those in less chaotic households (54). Others found that food insecurity was linked to children's social-emotional and cognitive outcomes in schools (55, 56). However, no prior studies examined how food insecurity and living conditions affected specific health behaviors such as physical activity, screen time, and sleep. When separating the analyses by weight status, our study found significant correlations between health behaviors and SDOHs (living conditions with screen time; food insecurity with physical activity) only in the NW group. This set of findings suggest the importance of considering weight status as a mitigating factor when determining the relationship between health behaviors and SDOHs in children from under-resourced communities.

This study makes several contributions to the literature of health disparities research. Firstly, our survey study followed a robust sequential sampling strategy to recruit a representative sample. We aimed to recruit participants through 18 schools but only 13 schools agreed to coordinate our recruitment, which might have affected the representativeness of the sample. Despite the difficulties of engaging schools and encouraging parents to complete the lengthy survey, we successfully executed the research plan and obtained a large diverse dataset that included children from a wide range of ADI and urbanicity categories, schools, grade and age levels, genders, races, and ethnicities. Secondly, we examined four impactful health behaviors and a few SDOHs as well as their relationships with explorative emphasis on weight status. The comprehensive study focus was important to understand the complex nature of health disparities and their determinants or correlates. Thirdly, we adopted questions from the MBQ-C to study different types of screen time across four dimensions (i.e., passive, interactive, sedentary, and standing) over weekdays and weekend days. Our study will pave the way for future research to comprehensively understand children's screen time by type, dimension, and duration as well as their health implications.

Nevertheless, several limitations of the study warrant attention. Firstly, given the cross-sectional design, the analyses and findings should not be interpreted as causal. Randomized controlled trials with interventions would be the more appropriate design to infer causal relationships. Secondly, like many studies based on a survey, our data were mostly self-reported by parent proxies and thus carry subjectivity bias and social desirability bias. Parent proxies as caregivers are commonly used to provide data for their young children in research like this. To mitigate these biases, we took a proactive approach during the data cleaning and processing process. We set a set of criteria to remove duplicates and outliers that would cause bias and errors to the dataset. Thirdly, the study may be subject to recruitment-related bias since only a portion of the contacted schools agreed to participate and assist our research team with data collection. The final sample may not fully reflect the broader population of children residing in the target communities. Fourthly, although group differences and associations were examined, the analyses did not incorporate adjustments for potential confounding characteristics, such as child age or sex that are known to shape health behaviors. Fifthly, data collection occurred across a long timeframe (October 2023–July 2024), where seasonal variation in health behavior by weather and daylight may have led to data fluctuations. Sixthly, the study integrated information derived at both the individual and community levels (e.g., household socioeconomic indicators and neighborhood ADI). The clustering effects were not empirically examined. Finally, the exclusion criterion in the sleep variable was applied only to total sleep time and some decisions were made by the researcher's judgement through inspection, where applying this cutoff may not have filtered outliers in the category of sleep during weekday and weekend days possible had impact on the representativeness of the final sample. Future research with a larger sample size at both individual and community levels should consider using multi-level modeling for data analysis.

## 5 Conclusion

This study identified differential patterns of health behaviors by weight status and SES, as well as the relationship between health behaviors and SDOHs with a secondary focus on the role of weight status. OWOB children showed a greater amount of screen time than NW children, and health behaviors were generally less favorable in those from under-resourced backgrounds. SDOHs such as household chaos, living conditions, and food insecurity showed significant correlations with health behaviors in children from under-resourced communities, and these correlations were slightly greater in NW children (vs. those with OWOB). Weight status played an important role in the relationship between health behaviors and SDOHs.

## Data Availability

The original contributions presented in the study are included in the article/Supplementary Material, further inquiries can be directed to the corresponding authors.

## Footnotes

^1^Godin-Shepard Leisure Score Index.

^2^Area Deprivation Index
