# Supplementary material for: Health behaviors and social determinants of health in children from under-resourced communities: does weight status play a role?
Source: Front Sports Act Living. 2026 Jan 5;7:1695539. doi: 10.3389/fspor.2025.1695539 (PMC12812871; doi:10.3389/fspor.2025.1695539)
Supplement: Supplementary file 1 [file Datasheet1.pdf]

Supplementary Material

Appendix

Table A1. Correlational Matrix for Variables (Health Behaviors and Environment; above diagonal = OWOB; below diagonal = NW) (*p<0.05, **p< 0.01, ***p< 0.001)

Variables	1	2	3	4	5	6	7	8	9	10	11	12	13	14	15	16	17	18	19	20	21
**1. Godin-Shepard LSI**	1	.54**	.01	.09	-.13	.03	.01	.04	.02	.07	-.06	.15	.04	.09	-.04	.15	.11	.14	-.11	.02	.01
**2. # of Active Days in a Week (days)**	.26**	1	-.02	.08	-.16	-.12	-.07	-.13	-.08	.03	-.17	.23**	.09	.02	.11	.08	.05	.09	-.14	.06	.05
**3.Passive screen time**	-.07	-.08	1	.80**	.40**	.26**	.21*	.16	.82**	.72**	.34**	-.07	.11	.09	.06	-.01	.04	-.07	-.16	-.04	-.09
**4.Sedentary passive screen time**	-.15	-.11	.79**	1	-.23*	.08	.20*	-.13	.59**	.86**	-.21*	.00	.05	.04	.03	-.06	-.03	-.07	-.12	-.04	-.04
**5.Passive screen time while standing**	.08	.01	.57**	-.05	1	.30**	.04	.46**	.44**	-.15	.88**	-.11	.10	.08	.05	.08	.11	.01	-.08	-.00	-.08
**6.Interactive screen time**	.13	-.01	.28**	.21*	.17	1	.81**	.65**	.76**	.49**	.54**	-.17	-.10	-.04	-.11	.12	.13	.06	.09	.09	.01
**7.Sedentary interactive screen time**	.07	-.00	.20	.25*	-.02	.89**	1	.09	.62**	.68**	.07	-.14	-.16	-.07	-.15	-.01	-.06	.06	.06	.07	.03
**8.Interactive screen time while standing**	.15	-.02	.23*	-.01	.39**	.48**	.03	1	.49**	-.05	.83**	-.12	.04	.04	.01	.21*	.30**	.03	.07	.07	-.03
**9.Total screen time**	.03	-.06	.81**	.64**	.47**	.79**	.67**	.44**	1	.77**	.54**	-.15	.02	.04	-.02	.06	.11	-.01	-.06	.02	-.05
**10.Total sedentary screen time**	-.05	-.07	.62**	.79**	-.04	.70**	.80**	.01	.83**	1	-.12	-.07	-.04	-.01	-.06	-.05	-.05	-.03	-.06	.00	-.02
**11.Total screen time while standing**	.13	-.00	.51**	-.04	.89**	.35**	.00	.77**	.54**	-.02	1	-.13	.08	.07	.04	.16	.23*	.02	-.01	.03	-.07
**12.Days with Regular Bed Routine**	.12	.07	-.26*	-.24*	-.09	-.13	-.09	-.13	-.25*	-.21*	-.13	1	.25**	.21*	.15	.04	-.02	.08	**-.24***	-.12	-.02
**13.Sleep Duration – Daily Average**	.05	.05	-.18	-.17	-.07	-.24*	-.24*	-.08	-.26*	-.26*	-.08	.17	1	.77**	.65**	.04	.03	.04	-.03	.02	-.02
**14.Sleep Duration on Weekday**	.27**	-.05	-.07	-.01	-.10	.08	.16	-.12	.01	.10	-.13	.17	.81**	1	.01	-.02	-.03	-.00	-.05	-.04	-.02
**15.Sleep Duration on Weekend Day**	.08	.03	-.04	-.03	-.01	.05	.09	-.05	.01	.04	-.03	.09	.63**	.06	1	.08	.08	.06	.00	.08	-.01
**16.Daily fruits and vegetables intake**	.17	-.07	-.03	-.13	.12	.12	.07	.17	.05	-.03	.14	.09	.08	.20*	.12	1	.88**	.83**	.12	.12	.06
**17.Daily fruits intake**	.08	-.07	-.02	-.14	.16	.11	.10	.06	.06	-.03	.14	.10	.10	.09	.07	.81**	1	.46**	.10	.11	.07
**18.Daily vegetables intake**	.19*	-.05	-.03	-.07	.04	.08	.02	.14	.03	-.03	.10	.06	.03	.23*	.13	.85**	.38**	1	.12	.10	.03
**19.Household Chaos**	-.04	-.01	.09	.13	-.02	.15	.17	.01	.15	.19	-.01	**-.19***	-.10	-.08	.08	-.02	.01	-.04	1	.27**	.35**
**20. Living condition**	.10	.03	.14	.13	.06	**.22***	.15	**.21***	**.22***	.17	.14	-.03	-.02	-.13	.13	-.01	.01	-.03	.27**	1	.45**
**21.Food Insecurity**	.19*	-.06	.01	.08	-.10	.17	.17	.05	.11	.16	-.04	-.13	-.08	-.10	.02	-.16	-.18	-.10	.26**	.50**	1
